# An eco-epidemiological study of Morbilli-related paramyxovirus infection in Madagascar bats reveals host-switching as the dominant macro-evolutionary mechanism

**DOI:** 10.1038/srep23752

**Published:** 2016-04-12

**Authors:** Julien Mélade, Nicolas Wieseke, Beza Ramasindrazana, Olivier Flores, Erwan Lagadec, Yann Gomard, Steven M. Goodman, Koussay Dellagi, Hervé Pascalis

**Affiliations:** 1 Centre de Recherche et de Veille sur les Maladies Emergentes dans l’Océan Indien (CRVOI), Plateforme de Recherche CYROI, 2 rue Maxime Rivière, 97490 Sainte Clotilde, La Réunion, France; 2 Université de La Réunion, UMR PIMIT “Processus Infectieux en Milieu Insulaire Tropical”, INSERM U1187, CNRS 9192, IRD 249, Plateforme de Recherche CYROI, Saint Denis, La Réunion, France; 3 Institut de Recherche pour le Développement (IRD), IRD – BP 50172, 97492 Sainte-Clotilde, La Réunion, France; 4 University of Leipzig, Department of Computer Science, Augustusplatz 10, D-04109 Leipzig, Germany; 5 Association Vahatra, BP 3972, Antananarivo 101, Madagascar; 6 Institut Pasteur de Madagascar, BP 1274 Ambohitrakely, Antananarivo 101, Madagascar; 7 UMR C53 CIRAD, Peuplements Végétaux et Bioagresseurs en Milieu Tropical, 7 chemin de l’IRAT, 97410 St Pierre, France; 8 Université de La Réunion, 15 Avenue René Cassin, 97400 Saint-Denis, France; 9 Field Museum of Natural History, 1400 S. Lake Shore Dr, Chicago, IL 60605-2496, USA

## Abstract

An eco-epidemiological investigation was carried out on Madagascar bat communities to better understand the evolutionary mechanisms and environmental factors that affect virus transmission among bat species in closely related members of the genus *Morbillivirus*, currently referred to as *Unclassified Morbilli-related paramyxoviruses* (*UMRVs*). A total of 947 bats were investigated originating from 52 capture sites (22 caves, 18 buildings, and 12 outdoor sites) distributed over different bioclimatic zones of the island. Using RT-PCR targeting the L-polymerase gene of the *Paramyxoviridae* family, we found that 10.5% of sampled bats were infected, representing six out of seven families and 15 out of 31 species analyzed. Univariate analysis indicates that both abiotic and biotic factors may promote viral infection. Using generalized linear modeling of *UMRV* infection overlaid on biotic and abiotic variables, we demonstrate that sympatric occurrence of bats is a major factor for virus transmission. Phylogenetic analyses revealed that all paramyxoviruses infecting Malagasy bats are *UMRVs* and showed little host specificity. Analyses using the maximum parsimony reconciliation tool CoRe-PA, indicate that host-switching, rather than co-speciation, is the dominant macro-evolutionary mechanism of *UMRVs* among Malagasy bats.

The transgression of the species barrier by pathogens moving from their natural host reservoir to infect a new host species (also referred to as host-switching, host-jumping or host-shifting), may induce an abortive infection in the few infected individuals of the new host, or trigger a short lived outbreak, or an epidemic^1^,^2^. Co-speciation and host-switching are the two main evolutionary mechanisms generating genetic diversity among micro-organisms. Both are long-term dynamic processes^3^, in contrast to co-evolution *sensu stricto*, which continuously acts on a short-time scale^4^. Co-speciation, the simultaneous speciation of the host and their parasites^3^,^5^,^6^,^7^, was considered for many years as the principal macro-evolutionary process generating viral diversity^8^,^9^,^10^,^11^. As convincing examples of co-speciation are rare, this mechanism has probably been overestimated. Host-switching refers to a new host-parasite combination that results from the shift of the parasite to a new host and its subsequent specialization, for example, under environmental selection pressure^12^. Colonization by a parasite of a phylogenetically closely related host species, often of the same genus or family, has proven to be the typical macro-evolutionary mechanism for RNA viruses^13^. An excellent example is the evolutionary history of *Hantavirus* and *Arenavirus*^14^,^15^, mostly shaped by multiple host-jumps, followed by adaptive processes within the new host, as demonstrated in bats and other operative host species^16^,^17^.

The often gregarious roosting behavior of bats and an assortment of different ecological parameters (e.g., climate, season, and migration) are important factors that can shape viral transmission dynamics, which subsequently act upon evolutionary processes^10^,^11^,^13^,^18^. Deciphering such mechanisms helps to understand how a virus hosted in wild animals can emerge as a pathogen in human populations^19^. For example, host-switching of *Ebola virus*, *SARS Coronavirus* or *Nipah virus* have led to major pandemics or epidemics in humans^2^,^20^,^21^.

*Paramyxoviridae* is a large and diverse viral family (Order: Mononegavirales) composed of single-stranded negative RNA viruses^22^. Newly recognized paramyxoviruses (PVs), named *Unclassified Morbilli-Related Viruses* (*UMRVs*), have recently been shown to infect small mammals around the world^23^, such as bats and terrestrial small mammals from the southwestern Indian Ocean (SWIO) islands^17^,^24^, including the biodiversity hotspot of Madagascar^25^. The island is divided into several unique bioclimatic zones, characterized by different meteorological regimes overlaid on elevation and underlying geology^26^, which in turn give rise to distinct vegetation types and highly endemic biotic communities.

After rodents, bats (order Chiroptera), constitute the most abundant, diversified, and geographically wide spread group of mammals in the world^27^. Genetic and fossil studies have estimated the basal split of placental mammals in the superorder Laurasiatheria from their ancestors at approximately 80–90 million years ago (Mya) and a diversification of bat families at approximately 62 Mya^28^. The Chiroptera of Madagascar are placed in eight different families and currently 45 species recognized, of which 36 species (80%) are endemic^29^,^30^,^31^; it is assumed that most originated from Africa. In certain cases, phylogenetic analyses provide evidence for recent periods of diversification. For example, Malagasy *Miniopterus*, a notably speciose genus, colonized the island from an African source population approximately between 4.5 and 2.5 Mya, followed by a second phase between 2.5 and 1 Mya^32^.

An important characteristic of Malagasy bat communities is that species co-occupy day roost sites in caves, buildings or tree cavities (often in forests) in different species combinations and varying numbers. Furthermore, certain bat species may have indirect contact with other wild, introduced or domestic animals, including synanthropic small mammals, which may imply contamination of shared common water sources or fruits by bat urine/saliva^29^. Considering the notable species diversity and high levels of endemism of Malagasy bats, as well as varying community structure and ecological conditions in which they occur, Madagascar provides an excellent context to study virus transmission in these animals. Herein, we examine the factors involved in interspecific transmission of *UMRVs* and try to unravel the macro-evolutionary mechanisms underlying genetic diversification in these viruses.

## Results

In total, 947 bats (867 insectivorous and 80 frugivorous), representing seven different families and 31 species, were collected at 52 sites in all six provinces of Madagascar: Antananarivo (n = 44 bats), Antsiranana (n = 125), Fianarantsoa (n = 178), Mahajanga (n = 207), Toamasina (n = 37), and Toliara (n = 356). The sampling sites included 22 different caves (n = 480 bats), 18 buildings (n = 290), and 12 different forested areas (n = 177). Thirty-one sites (n = 664 bats) contained at least two species and 21 sites (n = 283) were monospecific. The sampling sites were in different elevational zones, ranging from low (0 to 800 m, n = 40 sites), mid (801 to 1000 m, n = 6), and high (over 1000 m, n = 6), with 761, 101, and 85 bats collected in each zone, respectively. Seventeen sites were sampled in dry (n = 384 bats), 22 in sub-arid (n = 382), 11 in sub-humid (n = 144), and two in humid (n = 37) bioclimatic zones. Twenty-two sites (n = 377 bats) were visited during the summer (warm, wet) season and 30 sites (n = 570) during the winter (cool, dry) season.

**Figure 1:**
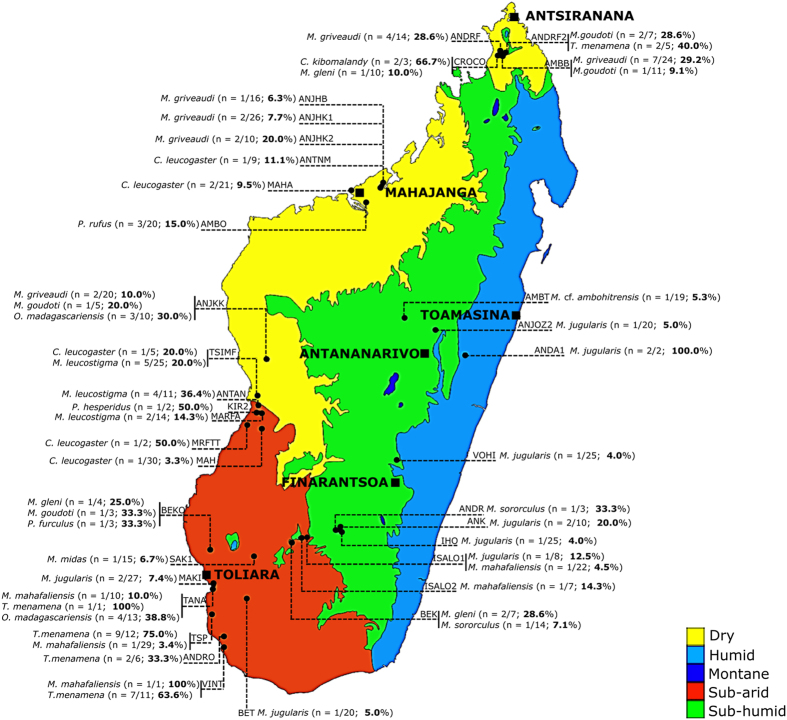
PVs detection rates among the sites sampled on Madagascar. Only sites containing positive bats are represented. Abbreviations refer to the names of sampling sites (e.g. ANDRF for “Andrafiabe”). n, numerator = the number of individuals that tested positive for PVs and denominator = the number of individuals tested. Provincial capitals are indicated by black squares. QGIS^46^, an open-source GIS software (http://qgis.osgeo.org/en/site/), was used to generate the map for visualizing bioclimatic regions of Madagascar proposed by Cornet^47^.

Ninety-nine of 947 bats (10.5%) tested positive for PVs by RT-PCR, giving a global infection rate of 11.1% in insectivorous bats and 3.8% in frugivorous bats (df (degrees of freedom) = 1; n = 947; χ^2^ *P* = 0.02). The infection rates varied according to province, from 4.5% in Antananarivo to 15.2% in Antsiranana (df = 5; n = 947; χ^2^, *P* = 0.01). The infection rates of PVs for bats living in caves, buildings, and forests were 12.9%, 7.9%, and 7.9%, respectively (df = 2; n = 947 χ^2^, *P* = 0.041). The fraction of sites hosting PV positive bats among the 31 multispecies sites and the 21 monospecific sites were 70.9% and 61.9%, respectively (df = 1; n = 947; χ^2^, *P* > 0.05). The infection rates for PV were 11.4% in multispecies sites and 8.1% in monospecific sites (df = 1; n = 947; χ^2^, *P* > 0.05). Infection rates at individual sites varied from 2.0% at ANJHB to 38.1% at VINT with no PV positive bat at 17 sites (n = 121) (see Fig. 1 for identification of sites and associated acronyms). At low, middle, and high elevation, the fraction of sites hosting PV positive bats was 67.5%, 83.3%, and 50.0% (df = 2; n = 947; χ^2^, *P* > 0.05), respectively, and the mean positive rates were 11.4%, 8.9%, and 3.5%, respectively (df = 2; n = 947; χ^2^, *P* > 0.05). In the humid, sub-humid, sub-arid, and dry bioclimatic zones, the percentages of sites hosting PV positive bats were 50.0%, 54.5%, 72.7%, and 70.6%, respectively (df = 2; n = 947; χ^2^, *P* > 0.05) and the mean positive rates were 5.4%, 6.3%, 12.0%, and 10.9%, respectively (df = 2; n = 947; χ^2^, *P* > 0.05). PV positive rates were 7.9% and 12.1% for bats captured during the summer and winter seasons, respectively (df = 1; n = 947; χ^2^, *P* = 0.038). Sites with *UMRVs* detection rates higher than 20.0% are indicated on Fig. 1.

Six of seven sampled bat families yielded PV positive individuals, with the exception being Hipposideridae, for which the only Malagasy species is *Hipposideros commersoni* (Table 1). The highest PV detection rate was in the family Rhinonycteridae (39.3%) and the lowest in the family Pteropodidae (3.8%) (df = 6; n = 947; χ^2^, *P* < 0.001). Half of the sampled species (16/32) contained PV positive individuals. The highest PV infection rate was in *Triaenops menamena* (n = 21/42; 50.0%) and the lowest in *Miniopterus mahafaliensis* (4/89; 4.5%) (df = 31; n = 947; χ^2^, *P* < 0.0001). Insectivorous species had significantly higher detection rates (96/867; 11.1%) than frugivorous species (3/80; 3.8%) (df = 1; n = 947; χ^2^, *P* = 0.02). No significant difference was found associated with sex and age classes, regardless of diet, habitat or site (χ^2^, *P* > 0.05).

**Figure 2:**
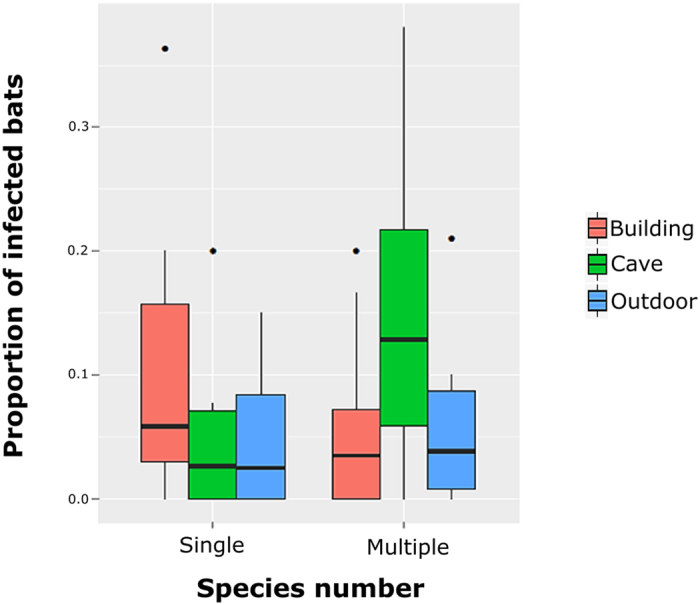
Proportion of infected bats depending on species diversity at each sampling site and the context of the where the samples were collected. Individual outlying data points are displayed as circles.

Model construction procedure lead to a binomial Generalized Linear Model (GLM) explaining individual infection based on seven different effects (Table 2). Among abiotic factors, Mean Annual Temperature (MAT) had an overall effect where, Mean Annual Rainfall (MAR) did not show any overall relationship with infection. However, relationships between rainfall and infection appeared different across multi- *versus* single-species sites with a quadratic effect observed for MAR. Habitat type and the multispecies characteristics did not show any significant effects, but showed marginal interaction. The multispecies sites show higher infection rates, compared to monospecific sites, for caves compared to buildings and forest capture sites (Fig. 2), reinforcing the important role of multispecies bat environments on PV infection. Diet was also associated with viral infection (Table 2), with higher infection among insectivorous bat species, whereas, age and sex did not show any significant relationships. Generalized Linear Mixed Model (GLMM) with species, locality, and province as random factors were tested separately and did not improve the fit, but models with family, species and locality failed to converge due to numerical issues in model estimation.

**Figure 3:**
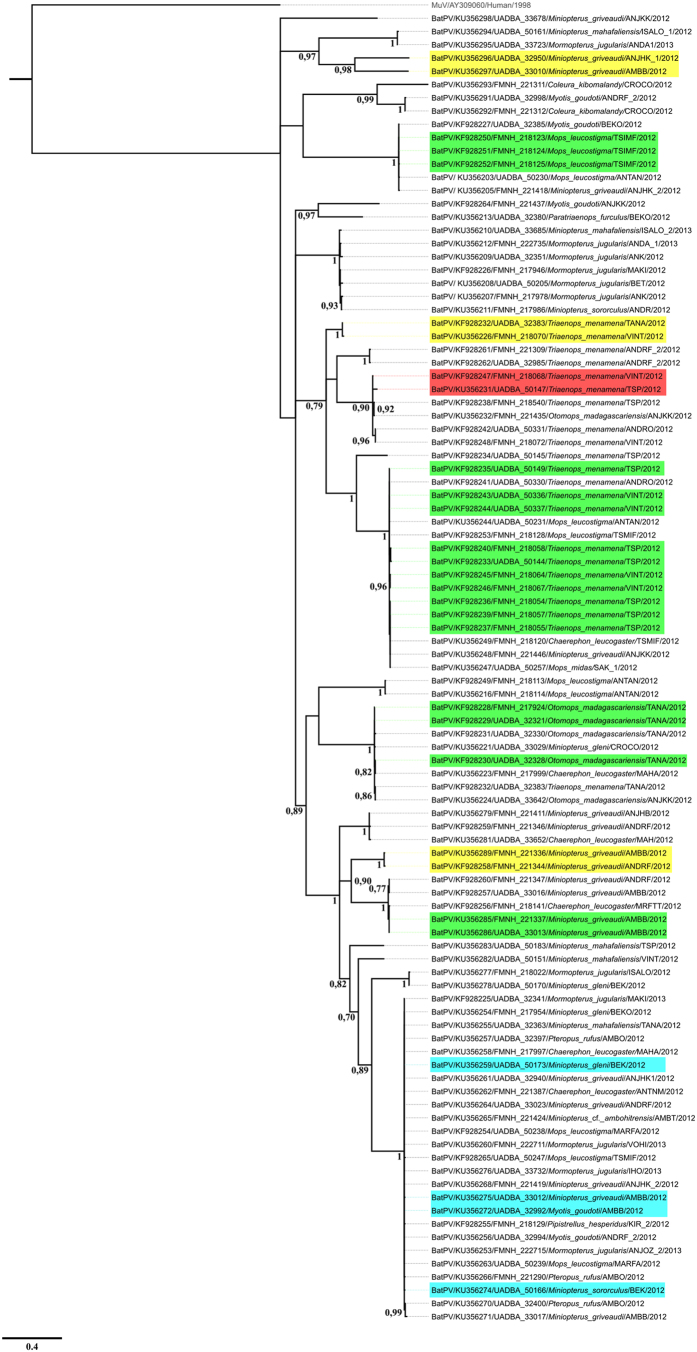
Phylogeny of the *UMRVs* detected in bats from Madagascar. A global phylogeny of 99 partial L-gene sequences calculated in 50,000,000 iterations in MrBayes with the GTR+G evolutionary model and a 10% burn-in rooted with a *Mumps* virus sequence (GenBank number AY309060). Only Bayesian with posterior probabilities >0.7 were represented. Host switching events were highlighted in blue and host-specificity for bats sharing the same sites in green. Bat species occurring at distant sites are highlighted in red. Bats living at distant sites and hosting with low level of *UMRVs* nucleotide similarity are highlighted in yellow.

We conducted a Bayesian analysis on the PV sequences generated from positive Malagasy bats together with PV GenBank sequences from Madagascar and elsewhere in the world. All new PV sequences presented in this study were identified as *UMRVs*, as they appeared more closely related to morbilliviruses^17^ (Supplementary Figure S1), than to any other genera of the *Paramyxoviridae* family. The *UMRV*s were characterized by a high level of genetic variability and nucleotide sequences varied from 62.0 to 100% sequence identity. Only two sequence pairs of the 99 that tested positive were identical. Although *URMVs* showed weak exclusivity to their bat host species, two phylogenetic patterns were identified: (*i*) closely related *UMRV* sequences were hosted by bat species and families that are phylogenetically closely related, particularly those occupying day roost sites in the same caves i.e., *Miniopterus griveaudi* and *Myotis goudoti* at AMBB; *Miniopterus gleni* and *Miniopterus sororculus* at BEK; (highlighted in blue in Fig. 3). This feature suggests that host-switching events might be favored by physical proximity between phylogenetically closely related bat taxa.

(*ii*) some degree of host-specificity for *URMVs* was found, with individuals of one host species having closely related *UMRVs*, independent of other individuals occurring at the same roost site, (i.e., *Triaenops menamena* at VINT TSP, and ANDRF2) highlighted in green, or distant sites, (i.e., *Triaenops menamena* at VINT and TSP), highlighted in red on Fig. 3.

In some cases, a correlation was observed between the distance separating capture sites and the degree of nucleotide sequence similarity of the infecting PVs across sites. More specifically, conspecifics living on distant sites host *UMRVs* that display lower level of nucleotide similarity than those infecting bats at sites in closer geographical proximity (i.e., *Triaenops menamena* at VINT and TANA, *Miniopterus griveaudi* at AMBB and ANJHK1, and *Miniopterus griveaudi* at ANDFR and AMBB) highlighted in yellow in Fig. 3. This suggests that increasing geographical distance favors virus genetic differentiation and/or low levels of virus migration between bat roosting sites.

**Figure 4:**
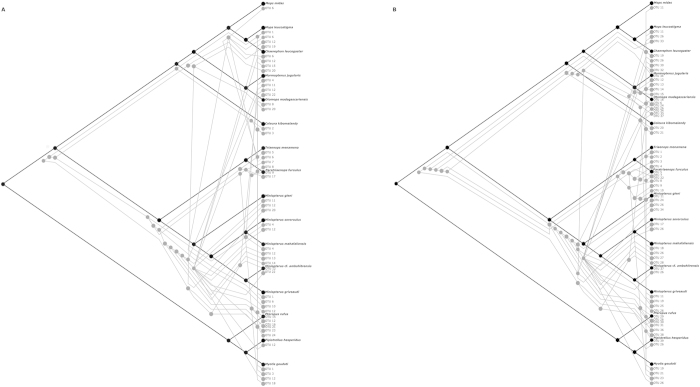
The first preferred reconstruction with the first best-cost model fit of the co-evolutionary history for the set of (**A**) 24 OTUs and (**B**) 39 OTUs and associated bat-species retrieved from CoRe-PA software. Host tree is represented in black; parasite tree is represented in grey.

**Figure 5:**
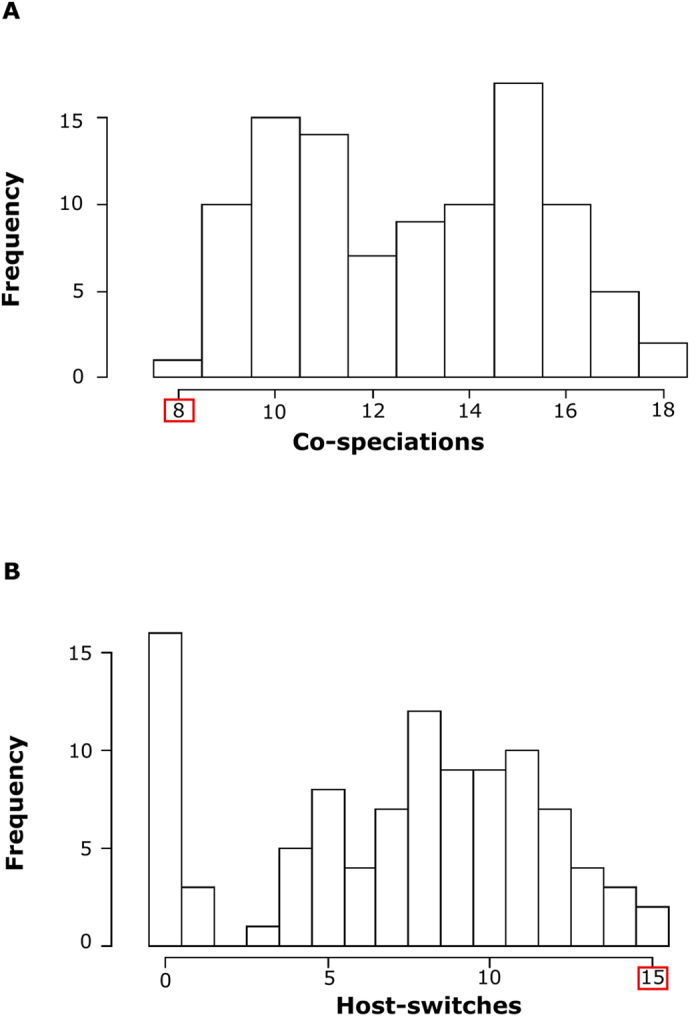
Frequency of co-speciation (**A**) and host-switches (**B**) events for the set of 24 OTUs obtained by random reconstructions. The number of co-speciation and host-switches events expected in the most parsimonious reconstructions by CoRe-PA, 8 and 15, respectively (framed in red; also in Table 3) were compared to the random reconstructions events below. The macro-evolutionary events showing lower random reconstruction events than expected (8 or 15) was determined as the predominant event.

Using CoRe-PA, we performed a consensus phylogram for both viruses and bats, presented along with their tanglegram depicting bat-virus associations (Fig. 4A,B). By evaluating 5000 random cost schemes, CoRe-PA computed the most parsimonious reconstruction and predicted the frequencies for co-evolutionary events, including co-speciation, host-switching, duplication, and sorting. For the generated 24 OTUs subset (Table 3a), the best quality value obtained was 0.256 for a solution with eight co-speciation events, 21 duplications, 52 sortings, and 19 host-switches. For the 39 OTUs subset, CoRe-PA produced 57 reconstructions (Table 3b), with a quality value of 0.25 and five co-speciation events, 33 duplications, 57 sorting, and 24 host-switches. Hence, for both sets, no clear evidence of co-speciation between *UMRVs* and bat species was found. The statistical analysis suggests fewer co-speciation events in the data set than expected by chance (99.0% of randomized data sets showed more than eight co-speciation events) but more host-switching events than expected (100% of randomized data sets showed less than 19 host-switching events) (Fig. 5A,B). Thus, notwithstanding the numerous identified duplication and sorting events, host-switching events appear to be the predominant aspect in the evolutionary history in *UMRVs* identified from Malagasy bats, as compared to co-speciation.

We quantified the degree of congruence between bats and *UMRVs* topologies, and the potential individual associations for each of the two OTU subsets. The hypothesis associated with independent speciation events could not be rejected by ParaFit (ParaFitGlobal = 38.62571; *P* = 0.067), for the 24 OTUs subset, whereas a significant overall pattern of association (ParaFitGlobal = 46.158; *P* = 0.002) was calculated for the 39 OTUs subset. Eight of 50 (16.0%) individual host-virus links were significant, based on a *P* < 0.05 for the 90.0% threshold, and 19 out of 60 (31.7%) for the 98% threshold. Tables S3a and S3b summarize the different associations of *UMRVs* with their respective hosts and the corresponding *P*-values for the two OTU subsets. Among the different bat species, *Triaenops menamena* was the most coupled species for both OTUs subsets, and *Miniopterus mahafaliensis* for the 39 OTUs subset. Depending on nucleotide identity, we observed a discrepancy of the global association signal, which is related to specificity increasing genetic variability by increasing the number of clades (i.e., increasing the nucleotide acid identity between sequences).

## Discussion

The overall *UMRV* infection rate in Malagasy bats was 10.5%, we also found that in some cases, that certain bat families or species showed higher PV detection rates. Four bat species had particularly high *UMRV* infection rates: *Triaenops menamena*, *Mops leucostigma, Miniopterus griveaudi*, and *Miniopterus gleni*. Except for the latter taxon, all were living at sites where substantial virus circulation was recorded (Fig. 1). Whether these species have higher susceptibility to PV infection cannot be discerned based on current data.

Moreover, statistical modeling demonstrated that environments supporting multiple species are positively associated to viral transmission, with a marginal effect of natural habitats (caves) being more prone to PV infection, whereas habitat type alone was not a significant predictor of infection. As previously reported, the spread of viruses between bat species is promoted by sympatric conditions, specifically multispecies day roost sites^10^. Other studies on bat rabies transmission demonstrated the importance of sympatric occurrence for viral infection^16^. The high detection rate in multispecies sites likely results from greater species diversity in caves^29^, inducing a proximity effect between individuals, which has been previously shown to promote virus transmission. Further work correlating rates of infection in caves as a function of bat density would help support this hypothesis; however, because of seasonal variation in bat density associated with population cycling and possible dispersal movements, this aspect will be difficult to document based on field studies. Certain climatic factors also seem to promote viral transmission: the probability of PV infection increased at localities with higher mean annual temperature, which favors infection compared to cooler regions. This result supports the importance of warmer temperature on viral transmission^18^. Whereas PV infection showed no overall relationship with rainfall, average rainfall conditions favored PV infection in multispecies sites, compared to drier/wetter conditions and to single-species sites. Further analyses need to be conducted to have a greater understanding of the role of climatic factors on infection. Finally, we also note that circulation of *UMRVs* seems to be much more active among insectivorous than frugivorous bat species, with only 3.8% of the latter tested positive. These results confirm previous studies conducted on SWIO islands^17^,^24^.

The bat-associated *UMRV* phylogeny underlines several points, particularly among the four taxa with the highest infection rates:

1. Bats collected sympatrically or some cases syntopically in the same day roost sites, for example, *Miniopterus griveaudi* and *Myotis goudoti*, belong to the families Miniopteridae and Vespertilionidae, respectively, host closely related viruses, suggesting that host-switching events occurred between these species/families;
2. Bat/virus co-phylogenies, suggest that co-speciation cannot explain the observed patterns. Host-switching is the predominant macro-evolutionary process. In either case, numerous reciprocal selection pressures that act over the short-term scale, such as the *sensu stricto* co-evolutionary process, also drive host-virus interactions. Indeed, micro-evolutionary aspects, including those implying selection, drift, and dispersal, result in intraspecific co-divergence of viruses^33^,^34^,^35^,^36^. Using CoRe-PA, we highlight the lack of congruence between bat and *UMRV* phylogenies. In previous studies it has been shown that a large number of phylogenies, set at the family level, including the *Paramyxoviridae*, are driven by this mode of macro-evolution^13^. Moreover, a considerable number of spillover events have been reported between rodents and bats^17^,^23^. Our phylogenetic analyses show that the same *UMRVs* infect different bat species or families, leading to the observed phylogenetic incongruence. This aspect can, at least in part be explained by the extremely rapid evolution of some RNA viruses, which as a consequence of their higher mutation rate^37^ generate large quasi-species virus populations, allowing for greater chances after a host-jump to adapt to a new host or, in other words, to promote a better adapted variant that can be sustained in the new host^38^. Examples of such macro-evolutionary processes, driven by host-switching, have been reported for *Puumala virus* and a *Hantavirus* detected in bats from northern Europe and for which no evidence of co-divergence was observed^39^. This scenario has also been cited for other hantaviruses and is probably a general rule for this viral family^14^,^40^;
3. A viral allopatric process, in which a virus speciates within a host species living in different geographical areas, and giving rise to independent evolution^12^,^13^. This may have occurred for *Triaenops menamena*, *Mops leucostigma*, and *Miniopterus griveaudi*; these three taxa have relatively broad distributions on Madagascar^26^. Interestingly, we could observe 7 major but phylogenetically distant viral clades with 5 or more closely related viruses detected in different bat species or families. This observation may suggest the circulation of 7 major *UMRVs* strains across Madagascar infecting a large host range. The CoRe-PA analysis indicates for these three species, 33 events of duplication. Duplication is a virus speciation event that occurs within the same host. This phenomenon can be the consequence of events that affect only the host, i.e., adaptive co-evolution, such as environmental adaptation. Such duplication events can be, for example, an immune pressure selection or virus specialization related to adaptation to different organs of the host. We also disproportionately found numerous duplication events in our analysis (21 duplications for the 24 OTUs and 33 duplications for the 39 OTUs subsets). This was anticipated, as CoRe-PA tends to place too many nodes from the virus tree near the root of the host tree. Consequently, whenever two descendant parasites (i.e., parasites that emerged straight from the same ancestral parasite) share the same host, a duplication event is predicted. One explanation could be associated with the capability of a virus within the host species to replicate independently. Different species of *Miniopterus* can be found roosting in strict syntopy and this close physical contact between related bat taxa may facilitate host-switching followed by mutation and duplication within the host. These sorting events might have multiple evolutionary causes and several hypotheses can explain these observations: (*a*) an ancient co-speciation event between the ancestor of the host and the virus, but the viral descendants subsequently went extinct; (*b*) an unidirectional and irreversible host-jump of the ancestral virus from one host to another; (*c*) no host-virus association ever existed between the virus and the respective sibling host;
4. As indicated by ParaFit Global test, significant associations were observed with the OTUs subsets (Tables S5a and S5b). We found significant linkage associations between species s uch as *Triaenops menamena* and both OTUs subsets. These results matched with our virus phylogeny analysis, which indicate some host-specificity, in particular for *Triaenops menamena*. We hypothesize that this association occurred after multiple host-switching events at some point in the past (macro-evolution) and continued in the form of a co-evolutionary adaptation (micro-evolution) inside the new host. Such phenomenon has also been reported in different coronaviruses^41^, for which co-evolution with bats seems to be the predominant evolutionary process. Since phylogenetically distant bat families hosted closely related *UMRVs*, the genetic distance between different groups of bats does not seem to be a major constraint for host-switching. Such results are important since host and virus traits determine the ability for a virus to infect a new host and host-switching events should *a priori* occur between phylogenetically closely related bat species on Madagascar. Besides, the occurrence of multiple interspecies transmissions, even to genetically distant host species, could be promoted by the existence of ubiquitous or alternative receptors for the virus^42^,^43^. It has been shown that genetic distances between bat species are a key factor for host-switching events^13^,^16^,^44^. However, our data also indicate that genetically closely related *UMRVs* infecting different bat species, sometimes occurring in geographically distant areas, may suggest the intervention of a probable vector, capable to connect these different populations^1^. Furthermore, except for regular bat foraging or dispersal movements, the black rat, *Rattus rattus*, introduced to Madagascar, has been identified as a significant reservoir of *UMRVs*^17^. This rodent might be the ideal candidate to play this spreading role and establishing epidemiological bridges between different species.

## Methods

### Fieldwork and sampling

This study used samples collected in the context of a long-term project to document the land vertebrates of Madagascar based on voucher specimens and for a variety of studies^26^. From February 2012 to March 2013, bats were captured in the six different provinces of Madagascar using harp traps, hand nets, and mist nets. Some *Pteropus* fruit bats were purchased in markets. Individual bats were identified to species using external and cranio-dental characters and comparison to museum specimens. For each animal, different parameters, including age, sex, and reproductive condition^45^ were recorded and this information deposited in DRYAD (http://dx.doi.org/10.5061/dryad.06g12).

Bat tissue samples were collected in the field and immediately stored in liquid nitrogen, then transferred to −80 °C storage upon arrival in the laboratory. The geographic ranges of the captured bat species were variable, with some having broad distributions nearly across the complete island and others distinctly more restricted. Several species, especially insectivorous bats, occur sympatrically in the same cave systems and in the same forest blocks, or synanthropically in human-built structures. Information on the species, province and specific collection locality, sampling season, geographic coordinates, elevation, habitat type, and the number of bat species found at each site and the associated species composition are presented in Tables S1 and S2. Mean climatic conditions of the sampling sites were extracted from the WorldClim database (http://www.worldclim.org/). We used the resolution proposed by WorldClim as 30 arc seconds (∼1 km). An open-source GIS software, QGIS^46^, was used to generate the map for visualizing Madagascar bioclimatic regions proposed by Cornet^47^.

### Ethics statement

Animals used in this study were manipulated in strict accordance with the guidelines for the handling of wild mammals^48^. All protocols strictly followed the terms of research permits and regulations and were approved by licencing authorities: Direction du Système des Aires Protégées et Direction Générale de l’Environnement et des Forêts and Madagascar National Parks under different research permits (n°194/12/MEF/SG/DGF/DCB.SAP/SCB, 067/12/MEF/SG/DGF/DCB.SAP/SCBSE, and 032/12/MEF/SG/DGF/DCB.SAP/SCBSE). Animals were captured, manipulated, and dispatched with thoracic compression following procedures accepted by the scientific community for the handling of wild mammals^48^. *Pteropus* were purchased in a market and were not physically collected by the research team in a natural setting. Euthanasia was used for *Pteropus* and not any other bat genera. All fieldwork conducted on Madagascar was before the creation and implementation of an institutional and/or licensing committee on the island to issue such clearances. A CITES permit from the Malagasy national authority was issued for *Pteropus* tissue export (n°243C-EA06/MG12) to CRVOI on La Réunion.

### Statistical procedures

Exploratory analyses were performed using Pearson chi-square (χ^2^) or Fisher’s exact tests in R software^49^ (95% confidence intervals with continuity correction). With the intent of identifying variables potentially correlated with *UMRV* infection, we performed a binomial GLM analysis^50^. We first visually inspected the relationships between variables (mean annual temperature [MAT], mean annual rainfall [MAR], habitat type), and “multi” a binary factor indicating whether a given site contained multiple (>2) or one species of bat. Graphic inspection suggested an overall effect of MAT and no effect of MAR. However, relationships suggested a linear interaction between MAR and habitat types, and a possible quadratic effect of rainfall within multi- *versus* single-species sites. Main and interaction effects were tested separately while accounting for the effects of other variables. We retained the best model according to Akaike Information Criterion (AIC). We then tested the effects of biotic variables (sex, diet, and age), on our best model, to determine a significance effect while accounting for the effects of abiotic factors. GLMM^51^ were constructed with unbalanced variables (i.e., province, localities, and species - related to non-homogenous sampling) set as random factors in order to be compared to the best GLM fit. Statistical analyses were conducted with R software package^49^.

### Sample screening

Approximately 1 mm^3^ of lung, kidney, and spleen collected from the same animal were pooled in DMEM medium and homogenized in a TissueLyser II (Qiagen, Hilden, Germany) for 2 min at 25 Hz using 3 mm tungsten beads. Total nucleic acids were extracted from the mixture supernatant using the viral mini kit v2.0 and an EZ1 BioRobot (Qiagen). cDNA products were generated via reverse transcription (cDNA kit, Promega, Madison, Wisconsin, USA). PVs were detected by a semi-nested PCR targeting part of the L-gene polymerase gene, designed as to detect *Respirovirus*, *Morbillivirus*, and *Henipavirus* (RMH)^52^. The 400–600 bp PCR amplified cDNAs were purified using the Qiagen PCR purification kit and cloned into the pGEM-T vector system (Promega). Cloned PCR products were sequenced by the Sanger method (Big Dye sequencing kit, ABI, Genoscreen, Lille, France) using M13 standard sequencing primers.

### Bioinformatics analysis

The sequences were first compared to the published sequences from the *Paramyxoviridae* and published *UMRVs* in GenBank (National Center for Biotechnology Information, Bethesda, Maryland, USA) online (www.ncbi.nih.gov) using BLASTn and BLASTx. The sequence quality of individual reads was assessed, and all sequences were processed using the Geneious Pro software package v7.1.8^53^. DNA sequences obtained from at least three independent bacterial clones were aligned to correct for most sequencing or PCR introduced errors. M13 Primer sequences were trimmed from the finalized sequences. The resulting partial sequences (~490 bp) of the L-gene polymerase gene were then aligned with Translation Alignment using the default ClustalW cost matrix in Geneious Pro software package. PVs sequences from bats reported in a previous study by Wilkinson *et al.*^17^ were used for phylogenetic analysis (GenBank number to KF928225 to KF928256 and to K928261 to KF928265). PVs and bat Cytochrome b (Cyt-b) sequences used for the present analyses were deposited in GenBank under the reference numbers in respectively Table S3 and Table S4. Information concerning amplified PV sequences is given in Table S3. In order to classify the detected new paramyxoviruses, viral family-level phylogenetic analyses were performed. A total of 209 partial L-gene paramyxovirus sequences collected in Genbank were used. Sequences were trimmed to remove any free end gaps or were entirely removed if the obtained alignment did not provide at least 462 bp of non-gap overlap. Internal gaps were permitted. The tree was performed in 5,000,000 iterations in MrBayes with the GTR+G+I evolutionary model and a 10% burn-in rooted with an *Aquaparamyxovirus* sequence (GenBank number EF646380). Genbank accession numbers used for each virus genera are indicated in Table S6.

A best-fit nucleotide substitution model of the alignment was determined using jModelTest^54^ with the Corrected Akaike Information Criterion (AICc)^55^, and the most appropriate one for *URMVs* from Malagasy bats was GTR+G. Phylogenetic trees were constructed using MrBayes v3.2^56^ employing a Bayesian Markov Chain Monte Carlo (MCMC) method, rooted with a *Mumps* virus sequence (GenBank number AY309060). A minimum of two independent runs were made, with four chains in each run, for a total of 50 000 000, sampling every 5000 generations. The first 5000 trees burn-in were discarded. The obtained effective sample size values (ESS) for each parameter were all superior to 200. Trees obtained after the convergence point were summarized and visualized by FigTree 1.4.2 (http://treebioedacuk/software/figtree).

Available full-length Cyt-b gene sequences corresponding to each bat species that were positive for *UMRV* infection were downloaded from GenBank. When Cyt-b sequences were not available for a given bat species, PCR using primers targeting the Cyt-b gene were performed^57^ to generate ~1140 bp sequences. All bat Cyt-b sequences were aligned, the GTR+I+G model was also the most appropriate substitution model, and the phylogenetic relationship among bat species were analyzed using RAxML 7.2.8 Geneious plugin^53^ using 1000 generations. Two subsets of operational taxonomic units (OTUs) were defined using Mothur^58^, and based on two genetic distance cutoffs (90.0% and 98.0%), generated 24 and 39 representative sequences, respectively.

To study the history of co-evolution of *UMRVs* with respect to their associated bat hosts, we performed event-based co-phylogenetic reconciliation, using the tool CoRe-PA^59^. CoRe-PA is an event-based maximum parsimony method, which attempts to construct the most parsimonious co-evolutionary history of hosts and associated parasites. A cost is assigned to each type of co-evolutionary event (co-speciation, host-switching, duplication, and sorting) and then, the parasite phylogeny is mapped onto the host phylogeny, while trying to minimize the total costs of all occurring events. In contrast to many other co-evolutionary software packages, CoRe-PA does not require an *a priori* assignment of a cost scheme. It has been shown that the results of such analyses strongly depend on the designed cost scheme, and choosing a biologically meaningful cost scheme in an *a priori manner* may be difficult^59^. CoRe-PA can assess several random cost schemes and evaluate these, based on the best fit with respect to the resulting reconstructions. In our study, we performed reconstruction between the phylogenetic trees of *UMRVs* and bats, using 5000 random cost schemes. Furthermore, to test statistical significance, we computed the reconstructions of 100 random data sets, considering the same phylogenetic trees for bats and *UMRVs* with different bat and virus associations. In this case, the formulated null hypothesis is that there are more co-speciation and less host-switching events in the data set, than compared to data sets with random host-parasite associations.

We quantified the degree of congruence between bat and *UMRVs* topologies, and the potential individual associations leading to a potential co-phylogenetic structure using a global-fit method, ParaFit^60^. The latter program tests the independence of host and symbiont genetic or patristic distances, and specifically herein, tests the hypothesis of evolution independence between bats and *UMRVs*, i.e., one partner randomly evolving with respect to the other. Statistical analyses were done using the R software package^49^.

## Additional Information

**How to cite this article**: Mélade, J. *et al.* An eco-epidemiological study of Morbilli-related paramyxovirus infection in Madagascar bats reveals host-switching as the dominant macro-evolutionary mechanism. *Sci. Rep.* **6**, 23752; doi: 10.1038/srep23752 (2016).

## Supplementary Material

Supplementary Information

Supplementary Table S1

Supplementary Table S2

Supplementary Table S3

Supplementary Table S4

## Figures and Tables

**Table 1:** Detection rates of *UMRVs* in bats from Madagascar.

Family	Species	Total positive/totaltested (%)	Grand total forfamily
Emballonuridae	*Coleura kibomalandy*	2/6 (33.3)	2/9 (22.2)
	*Paremballonura tiavato*	0/3	
Hipposideridae	*Hipposideros commersoni*	0/27	0/27
Miniopteridae	*Miniopterus aelleni*	0/7	30/289 (10.4)
	*Miniopterus* cf. *ambohitrensis*	1/19 (5.3)	
	*Miniopterus gleni*	4/22 (18.2)	
	*Miniopterus griffithsi*	0/7	
	*Miniopterus griveaudi*	18/116 (15.5)	
	*Miniopterus mahafaliensis*	4/89 (4.5)	
	*Miniopterus majori*	0/7	
	*Miniopterus sororculus*	2/22 (9.1)	
Molossidae	*Chaerephon atsinanana*	0/34	36/406 (8.9)
	*Chaerephon leucogaster*	6/94 (6.4)	
	*Mops leucostigma*	11/68 (16.2)	
	*Mops midas*	1/19 (5.3)	
	*Mormopterus jugularis*	12/152 (7.9)	
	*Otomops madagascariensis*	7/39 (17.9)	
Pteropodidae	*Eidolon dupreanum*	0/11	3/80 (3.8)
	*Pteropus rufus*	3/20 (15.0)	
	*Rousettus madagascariensis*	0/49	
Rhinonycteridae	*Paratriaenops furculus*	1/14 (7.1)	22/56 (39.3)
	*Triaenops menamena*	21/42 (50.0)	
Vespertilionidae	*Hypsugo bemainty*	0/2	6/80 (7.5)
	*Myotis goudoti*	5/48 (10.4)	
	*Neoromicia malagasyensis*	0/2	
	*Neoromicia matroka*	0/4	
	*Neoromicia robertsi*	0/1	
	*Pipistrellus* cf. *hesperidus*	0/8	
	*Pipistrellus hesperidus*	1/11 (9.1)	
	*Pipistrellus raceyi*	0/3	
	*Scotophilus marovaza*	0/1	
Grand total			99/947 (10.5)

Numerator of individuals that tested positive for PVs over total number of individuals tested, corresponding percentage of positivity given in parentheses.

**Table 2:** Summary of the binomial GLM on individual infection (n = 947).

Effect	Df	Deviance	F value	Pr (>F)
MAT	1	604.1	9.166	0.002**
Habitat	2	598.9	0.4938	0.61
Multi	1	605.7	11.65	0.0006***
Diet	1	602.6	6.803	0.009**
Habitat:Multi	2	601.3	2.423	0.0891^^^
Multi:MAR	2	604.6	5.006	0.007**
Multi:MAR^2^	2	602.3	3.142	0.043*
Residuals		598.2		

The model was selected after inspection of bivariate relationships and interactions. Because of unbalance, type III sums-of-squares were used to test the effects. MAT: Mean Annual Temperature, Habitat: habitat type, Multi: multispecies/monospecific site, MAR: mean annual rainfall, Df: degrees of freedom associated with the effect, Deviance: deviance of the model, F value: value of Fisher statistics for the different effects, Pr (>F): *P* values associated with the tests. Symbols for *P* values as follows: ^<0.1, *<0.05, **<0.01, ***<0.001.

**Table 3:** Results for event base co-phylogeny obtained with CoRe-PA and number of the different events for sets of (A) 29 OTUs and (B) 39 OTUs.

OTUs	Reconstruction	(q)	Frequency of events				Total cost				
			Co-speciation	Sorting	Duplication	Host-switch	Co-speciation	Sorting	Duplication	Host-switch	Total
A	1A	0.256	8	52	21	19	0.227	0.064	0.146	0.564	18.91
	2A	0.277	7	47	21	20	0.241	0.071	0.162	0.526	18.93
	3A	0.286	10	47	18	20	0.171	0.076	0.187	0.566	19.96
B	1B	0.25	5	57	33	21	0.22	0.07	0.13	0.58	21.52
	2B	0.26	5	51	32	22	0.23	0.08	0.13	0.56	21.84
	3B	0.28	6	63	33	20	0.15	0.05	0.08	0.7	21.1
