# Supplementary material for: An eco-epidemiological study of Morbilli-related paramyxovirus infection in Madagascar bats reveals host-switching as the dominant macro-evolutionary mechanism
Source: Sci Rep. 2016 Apr 12;6:23752. doi: 10.1038/srep23752 (PMC4828640; doi:10.1038/srep23752)
Supplement: Supplementary Information [file srep23752-s1.pdf]

Supplementary Information

An eco-epidemiological study of Morbilli-related paramyxovirus infection in Madagascar bats reveals host-switching as the dominant macro-evolutionary mechanism.

Julien Mélade[1,2,3], Nicolas Wieseke[4#], Beza Ramazindrazana[1,2,3,5,6#], Olivier Flores[7,8$], Erwan Lagadec[1,2,3$],Yann Gomard[1,2,3$], Steven M. Goodman[5,9], Koussay Dellagi[1,2,3] & Hervé Pascalis[1,2,3*]

[#,$] Contributed equally.

[*] Corresponding author: Hervé Pascalis: Université de La Réunion, UMR PIMIT "Processus Infectieux en Milieu Insulaire Tropical", INSERM U1187, CNRS 9192, IRD 249, Plateforme de Recherche CYROI, Saint Denis, La Réunion, France. E-mail: herve.pascalis@ird.fr. Tel: +262 938835

[1] Centre de Recherche et de Veille sur les Maladies Emergentes dans l'Océan Indien (CRVOI), Plateforme de Recherche CYROI, 2 rue Maxime Rivière, 97490 Sainte Clotilde, La Réunion, France.
[2] Université de La Réunion, UMR PIMIT "Processus Infectieux en Milieu Insulaire Tropical", INSERM U1187, CNRS 9192, IRD 249, Plateforme de Recherche CYROI, Saint Denis, La Réunion, France.
[3] Institut de Recherche pour le Développement (IRD), IRD – BP 50172, 97492 Sainte-Clotilde, La Réunion.
[4] University of Leipzig, Department of Computer Science, Augustusplatz 10, D-04109 Leipzig, Germany.
[5] Association Vahatra, BP 3972, Antananarivo 101, Madagascar.
[6] Institut Pasteur de Madagascar, BP 1274 Ambohitrakely, Antananarivo 101, Madagascar.
[7] UMR C53 CIRAD, Peuplements Végétaux et Bioagresseurs en Milieu Tropical, 7 chemin de l'IRAT, 97410 St Pierre, France.
[8] Université de La Réunion, 15 Avenue René Cassin, 97400 Saint-Denis, France.
[9] Field Museum of Natural History, 1400 S. Lake Shore Dr, Chicago, IL 60605-2496, USA.

- Figure S1: Phylogeny of all sequences belonging to the *UMRV* phylogroup.
- Table S4: Bats cytochrome b sequences data set.
- Table S5: Test of host-parasite co-evolution using global fit methods ParaFit.

Figure S1. Phylogeny of all sequences belonging to the *UMRV* phylogroup. A global phylogeny of 308 partial L-gene sequences calculated in 10,000,000 iterations in MrBayes with the GTR + G + I evolutionary model and a 10% burn-in rooted with an *Aquaparamyxovirus* sequence (GenBank number EF646380). All Malagasy bat paramyxoviruses sequences obtained within this study fell within group of Unclassified Morbillivirus-Related viruses. Genbank accession numbers used for each virus genera are indicated in Table S6.

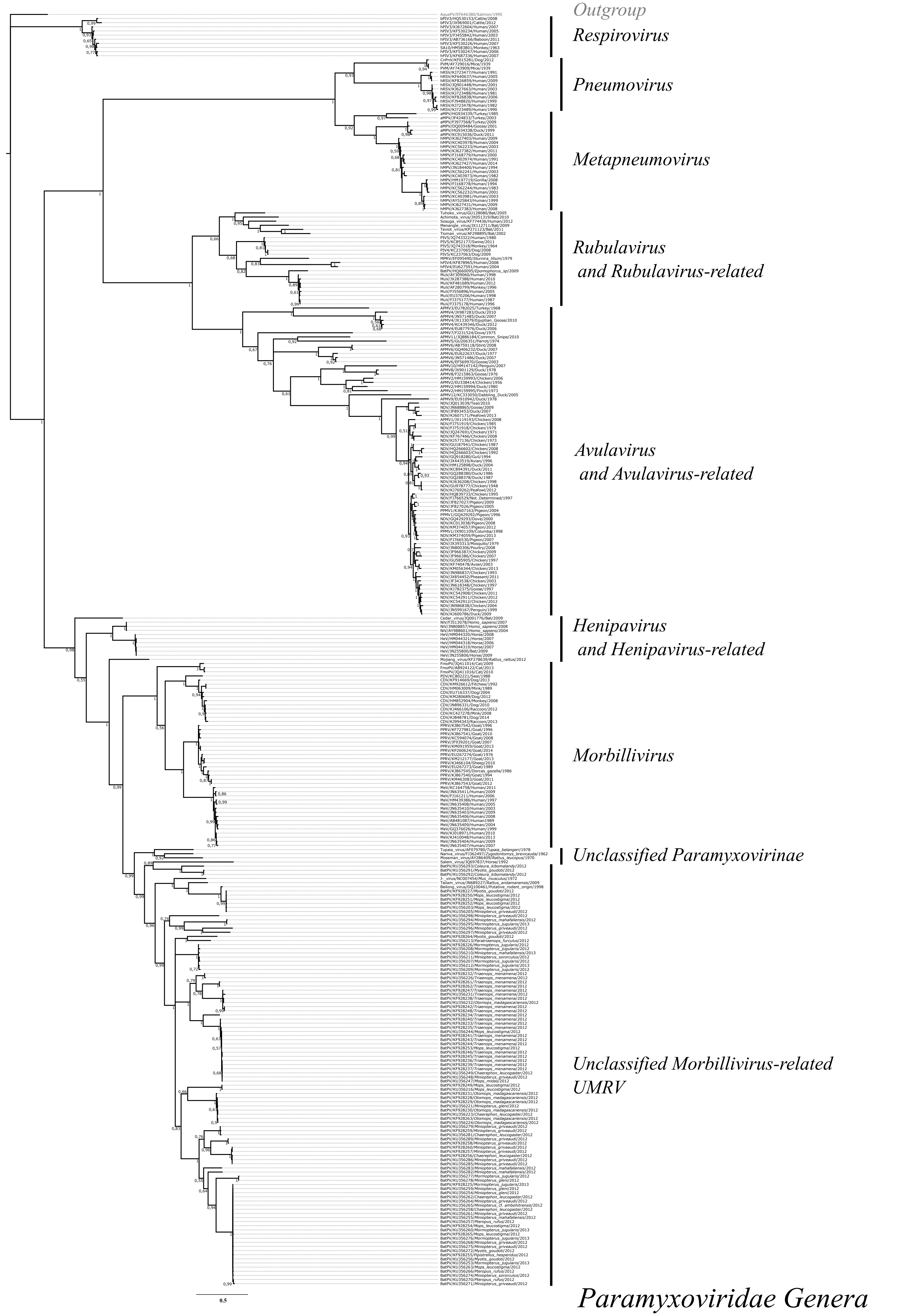
Outgroup
Respirovirus
Pneumovirus
Metapneumovirus
Rubulavirus
and Rubulavirus-related
Avulavirus
and Avulavirus-related
Henipavirus
and Henipavirus-related
Morbillivirus
Unclassified Paramyxovirinae
Unclassified Morbillivirus-related
UMRV
0.5
Paramyxoviridae Genera

Table S4. Bats cytochrome b sequences data set. Sequence generated as part of this study is underlined.

Bat family	Bat species	Cytochrome b Genebank accession number
Emballonuridae	*Coleura kibomalandy*	JQ710749
Miniopteridae	*Miniopterus mahafaliensis*	FJ383160
	Miniopterus gleni	FJ383146
	Miniopterus griveaudi	FJ232793
	Miniopterus cf. *ambohitrensis*	DQ899767
	Miniopterus sororculus	JF440282
Molossidae	*Chaerephon leucogaster*	KF193638
	Mops leucostigma	FJ546238
	Mops midas	EF474039
	Mormopterus jugularis	KR606332
	Otomops madagascariensis	EF216372
Rhynonicteridae	*Paratriaenops furculus*	KU361218
	Triaenops menamena	KR636334
Pteropodidae	*Pteropus rufus*	AB085732
Vespertilionidae	*Myotis goudoti*	GU116764
	Pipistrellus hesperidus	JX276312

Table S5. Test of host-parasite co-evolution using global fit methods ParaFit for the (a) 24 OTUs and (b) 39 OTUs. Respective significant *P* values for each association of bats and associated OTUs configurations are listed.
Host correspondence: N° 6: *Coleura kibomalandy*; N° 7: *Triaenops menamena*; N° 8: *Paratriaenops furculus*; N° 9: *Miniopterus gleni*; N° 10: *Miniopterus sororculus*; N° 11: *Miniopterus mahafaliensis*; N° 12: *Miniopterus manavi*; N° 13: *Miniopterus griveaudi*.

A)

Association	Host	Parasite	F1.stat	*P*, F1.stat	F2.stat	*P*, F2.stat
17	6	2	6.97370126	0.017	9.855293e-03	0.017
18	6	3	6.23723119	0.010	8.814507e-03	0.010
19	7	5	2.41905999	0.019	3.418636e-03	0.019
20	7	6	2.56628512	0.017	3.626696e-03	0.017
21	7	7	1.78432970	0.006	2.521630e-03	0.006
22	7	8	2.58266303	0.006	3.649841e-03	0.006
23	7	9	2.55353489	0.009	3.608677e-03	0.009
24	8	17	1.16336572	0.020	1.644078e-03	0.019
31	11	12	2.10816977	0.053	2.979283e-03	0.049

B)

Association	Host	Parasite	F1.stat	*P*, F1.stat	F2.stat	*P*, F2.stat
21	6	20	4.3861140	0.025	0.0095795508	0.018
22	6	21	4.0099203	0.014	0.0087579199	0.011
23	7	1	2.1418077	0.001	0.0046778437	0.001
24	7	2	2.1984815	0.001	0.0048016228	0.001
25	7	3	3.0098937	0.001	0.0065737985	0.001
26	7	4	3.5152171	0.002	0.0076774567	0.002
27	7	5	3.4920883	0.001	0.0076269419	0.001
28	7	7	3.6222159	0.002	0.0079111489	0.002
29	7	8	3.4558980	0.001	0.0075479000	0.001
30	7	9	3.8069554	0.001	0.0083146317	0.001
31	7	10	3.9627150	0.001	0.0086548205	0.001
32	7	11	3.8670228	0.001	0.0084458228	0.001
35	9	26	2.1162713	0.033	0.0046220705	0.030
38	10	26	2.3503911	0.033	0.0051334031	0.029
40	11	26	2.5833431	0.013	0.0056421850	0.009
41	11	27	2.2050280	0.039	0.0048159209	0.034
42	11	28	2.1135156	0.041	0.0046160520	0.037
44	12	26	2.3814991	0.023	0.0052013449	0.018
48	13	26	2.3268800	0.022	0.0050820533	0.021
